# Role of P2X7R in Retinal Diseases: A Review

**DOI:** 10.1002/iid3.70203

**Published:** 2025-05-21

**Authors:** Chunli Li, Binsheng Wang

**Affiliations:** ^1^ Department of Ophthalmology The Second Hospital of Lanzhou University Lanzhou Gansu China; ^2^ Department of General Surgery The First Hospital of Lanzhou University Lanzhou Gansu China

**Keywords:** age‐related macular degeneration, diabetic retinopathy, P2X7R, retinal, retinitis pigmentosa

## Abstract

**Background:**

P2X purinoceptor 7 receptor (P2X7R) is an ATP‐gated ion channel that, upon activation by ATP, triggers the release of inflammatory mediators and induces apoptosis in cells. This channel plays a crucial role in the onset and progression of various diseases. Recently, there has been a growing body of research focused on the function of P2X7R receptors in ophthalmic conditions, particularly concerning retinal diseases such as age‐related macular degeneration, diabetic retinopathy, and retinitis pigmentosa.

**Objective:**

This article is to provide a comprehensive review of the advancements in the study of P2X7R and its association with retinal diseases, elucidating its role in these conditions and identifying potential avenues for future research.

**Methods:**

Electronic databases, including PubMed, Web of Science, and Wan fang Data were searched for relevant literature. The following keywords were used: “P2X7R”, Age‐related macular degeneration”, “Diabetic retinopathy”, “Retinitis pigmentosa”. Both preclinical and clinical studies were included to provide a holistic understanding of P2X7R's role in retinal pathology.

**Results:**

P2X7R activation exacerbates retinal diseases by promoting inflammation and apoptosis. However, its role in disease progression and homeostasis complicates therapeutic targeting, highlighting the need for selective inhibitors and further research into its context‐dependent functions.

**Conclusion:**

P2X7R plays a critical role in the pathogenesis of retinal diseases. At the same time, preclinical studies suggest that P2X7R inhibition holds promise as a therapeutic strategy. Future research should focus on developing selective P2X7R inhibitors, elucidating the receptor's role in different disease stages, and identifying biomarkers to guide personalized treatment. Addressing these challenges will be essential for translating P2X7R‐targeted therapies into clinical practice and improving outcomes for patients with retinal diseases.

## 1 Introduction

With the ongoing advancements in biomedical technology, research on retinal diseases has become increasingly comprehensive. P2X7R, an ion channel protein that is widely distributed across cell membranes, has garnered significant attention in the field of retinal disease research in recent years [1, 2, 3]. The distinctive structure of P2X7R is pivotal in processes such as signal transduction, cell apoptosis, and inflammatory responses, all of which are closely linked to the pathogenesis of retinal diseases. Therefore, a review of the research progress regarding P2X7R and retinal‐related diseases will deepen our understanding of the underlying mechanisms and offer novel insights and strategies for clinical treatment.

P2X7R is a nonselective cation channel protein that belongs to the P2X subfamily. It is predominantly expressed on cell membranes and is involved in the exchange and signal transduction of ions both intracellularly and extracellularly [4]. In the retina, P2X7R is widely distributed among neurons and glial cells in both the inner and outer synapse‐rich layers, as well as in the nuclear layers of the retina [5], playing a crucial role in regulating neurotransmitter release and neuronal excitability. In terms of ATP signaling, ATP in the retina activates P2X7R, modulating calcium ion (Ca²⁺) influx, which influences neuronal activity and signal transmission. Additionally, P2X7R may be involved in the regulation of synaptic plasticity in the retina, thereby affecting the processing of visual information [5, 6]. As research on P2X7R advances, numerous studies have increasingly demonstrated a significant correlation between P2X7R and the onset and progression of various retinal diseases. For instance, in age‐related macular degeneration, elevated expression levels of P2X7R may contribute to the apoptosis and dysfunction of retinal cells [7]. In the context of diabetic retinopathy, abnormal expression of P2X7R could exacerbate inflammatory responses and further compromise retinal cell integrity. Furthermore, P2X7R is also implicated in the pathogenesis of retinal pigmentary degeneration and optic neuritis [8].

In this paper, we systematically summarize the role of P2X7R in retinal diseases, including age‐related macular degeneration (AMD), diabetic retinopathy (DR), and retinitis pigmentosa (RP). Our objective is to highlight its involvement in inflammatory processes, apoptosis, ion channel signaling, and retinal degeneration. Additionally, we will discuss the potential of P2X7R as a therapeutic target and evaluate the challenges associated with translating preclinical findings into clinical applications. By identifying gaps and controversies in current research, this review seeks to provide valuable insights for future studies and therapeutic development.

## 2 Literature Search

We conducted a literature search in PubMed, Web of Science, and Wan fang Data using keywords “P2X7R”, “Age‐related macular degeneration”, “Diabetic retinopathy”, and “Retinitis pigmentosa”. The initial screening process involved excluding irrelevant studies by reviewing titles and abstracts. This was followed by a full‐text review for further refinement, ultimately including only those studies that met the established criteria. We then categorized the literature according to the role of P2X7R in retinal diseases and extracted key data. In the presentation of results section, we summarized the mechanisms of P2X7R in various diseases using tables. Additionally, we provided a narrative synthesis organized thematically within the manuscript.

## 3 P2X7R

The P2X receptor family comprises seven subtypes (P2X1 to P2X7R), each characterized by a unique transmembrane structure that allows for the rapid transduction of extracellular ATP signals into intracellular responses [9]. Short‐term activation of the P2X7 receptor by ATP ( < 10 s) leads to rapid and reversible channel openings, permitting the passage of Na⁺, K⁺, and Ca²⁺ ions. Under normal physiological conditions, the presence of P2X7 receptors contributes to cell survival, regulates immune cell activity, and modulates inflammatory responses. However, in hypoxic or ischemic environments, elevated ATP concentrations, bacterial products, and inflammatory mediators increase the number of P2X7 receptors and heighten their sensitivity to ATP. Prolonged stimulation of these receptors results in the formation of large pores in the plasma membrane, allowing the uptake of cationic molecules up to 900 Da. This process further promotes the release of inflammatory mediators and ultimately leads to cell death [10]. Among these, P2X7R has garnered significant attention due to its high expression levels in immune cells and its pivotal role in activation, inflammatory responses, and apoptosis [11]. Furthermore, P2X7R has been implicated in the pathogenesis of various diseases, including autoimmune disorders, neurodegenerative diseases, and certain cancers [12, 13, 14, 15].

The P2X7R receptor is a trimeric protein characterized by an amino terminal, an extracellular domain, and a carboxy terminal. Each of these components contains two membrane‐spanning helices and a substantial extracellular domain that serves as the ATP binding site [4]. Upon ATP binding, the receptor undergoes conformational alterations that result in the opening of an ion channel. This process allows the influx of cations, such as Ca²⁺ and Na⁺, into the cell while simultaneously facilitating the efflux of K⁺ [16, 17]. This ion flux is essential for the initiating intracellular signaling cascades, which include the activation of various kinases and the release of pro‐inflammatory cytokines.

P2X7R is predominantly expressed in various immune cell types, including monocytes, macrophages, and T cells, plays a crucial role in mediating immune responses [4, 18]. Specifically, P2X7R is implicated in the formation of the NLRP3 inflammasome, which is essential for the production of pro‐inflammatory cytokines such as IL‐1β and IL‐18 [19, 20, 21]. This pathway is particularly significant in the context of chronic inflammation and autoimmune disorders. Recent studies indicate that P2X7R is vital for maintaining the homeostasis of the retinal microenvironment [2, 22, 23]. As a cation channel, P2X7R facilitates intercellular communication among retinal cells, which is essential for visual signal processing and the overall health of the retina. Furthermore, the modulation of ion flow through P2X7R is critical for preserving the stability of the retinal environment and supporting normal physiological functions [24].

Following retinal injury, activated immune cells, including macrophages and microglia, secrete a variety of bioactive molecules, such as ATP, which interact with P2X7R receptors. This interaction facilitates an increased flow of ions, initiating a cascade of inflammatory responses that may either support tissue repair or, conversely, contribute to retinal degeneration. The dual functionality of P2X7R highlights its potential as a therapeutic target; while it can mediate protective mechanisms in the aftermath of injury, excessive or dysregulated activation of P2X7R may lead to detrimental outcomes, including chronic inflammation and neuronal cell death.

## 4 P2X7R and Retinal Diseases

### 4.1 Diabetic Retinopathy

Diabetic retinopathy is a common ocular complication among individuals with diabetes, characterized by alterations in both the structure and function of the retina. As diabetes progresses, the deterioration of retinal integrity can lead to a significant decline in visual acuity and, in severe cases, result in blindness [25]. Studies suggest that elevated expression levels of P2X7R are observed in diabetic patients, which may be associated with the inflammatory response and immune responses triggered by diabetes [26, 27]. Additionally, P2X7R plays a critical role in the apoptotic processes of retinal neurons, thereby contributing to the onset and progression of neurodegenerative diseases. P2X7R is associated with retinal vasculopathy, which may precipitate vascular inflammatory responses and subsequent vascular damage [28]. Diabetes increased the vulnerability of retinal microvessels to sustained P2X7R activation, leading to the formation of large transmembrane pores and increased apoptosis and cell death [29]. Research has shown that the CD40‐ATP‐P2X7R pathway not only exacerbates inflammation but also induces retinal endothelial cell death, a key event in the development of capillary degeneration and retinal ischemia [30, 31]. Hyperglycemia triggers increased expression and opening of connexin 43 hemichannels on the cell membrane, resulting in elevated extracellular ATP levels. Subsequently, the increased extracellular ATP binds to P2X7R and directly activates the NLRP3 inflammasome, thereby initiating pyroptosis in HRMECs [32, 33].

The activation of the P2X7R receptor may exacerbate the inflammatory response associated with diabetic retinopathy [30]. Activated macrophages and immune cells release a variety of bioactive molecules, including proteins associated with the P2X7R channel [2, 34]. Following P2X7R activation, there is an enhancement in the release of inflammatory mediators, such as the cytokines IL‐1β, IL‐6, and TNF‐α [19]. Furthermore, P2X7R activation enhances the expression of adhesion molecules, such as intercellular adhesion molecule‐1 (ICAM‐1) [35], which facilitates the adhesion and infiltration of inflammatory cells [36]. These bioactive substances interact with P2X7R receptors to promote intracellular ion flow, thereby contributing to the inflammatory response. Research indicates that abnormal expression of P2X7R may lead to the apoptosis of retinal neurons and the exacerbation of neurodegenerative diseases, potentially linked to P2X7R‐mediated Ca^2+^ influx and neuronal death pathways [37]. Additionally, P2X7R may be implicated in the vascular damage observed in DR, including the production of nitric oxide (NO) and reactive oxygen species (ROS) [38]. The activation of P2X7R may further promote vascular inflammatory reactions.

Molecules associated with vascular endothelial cell injury, such as Vascular Endothelial Growth Factor (VEGF) [28], suggest that the P2X7R receptor may influence the growth and repair mechanisms of vascular endothelial cells. Inhibition of P2X7R may mitigate blood‐retinal barrier (BRB) dysfunction induced by hyperglycemic conditions [39, 40]. The retinal vasculature in diabetic patients frequently experiences complications such as narrowing, occlusion, and leakage, which can lead to retinal ischemia and hypoxia, thereby exacerbating retinal damage [27]. Aberrant expression of P2X7R may amplify the extent of vascular inflammation and injury. The P2X7R receptor is integral to the progression of diabetic retinopathy. By modulating the expression and functionality of P2X7R, it may be possible to protect the retina from damage, decelerate the advancement of neurodegenerative diseases, and enhance vascular function.

Although numerous studies have demonstrated that P2X7 receptor inhibitors can delay the progression of diabetic retinopathy, some research indicates that P2X7R may play a beneficial role in clearing pathogen infections during infectious diseases [41, 42]. This presents certain challenges. In the future, it may be essential to further investigate the role of P2X7R at various stages of diseases and develop biomarkers based on P2X7R expression or activity to guide personalized treatment. The function of P2X7R may differ across various diseases (or even at different stages of the same disease), highlighting the need for the development of disease‐specific intervention strategies.

### 4.2 Age‐Related Macular Degeneration

Age‐related Macular Degeneration (AMD) is a common retinal condition associated with the aging process. It is characterized by the deterioration of central vision, alterations in the retinal pigment epithelium, and the formation of abnormal subretinal deposits [43, 44]. These pathologies symptoms such as blurred vision and distortion of straight lines, which may ultimately result in progressive vision loss and potential blindness [45]. At the molecular biological level, the progression of AMD is closely associated with the expression and function of the P2X7R [22, 46]. P2X7R is a nonselective cation channel that plays a critical role in retinal cellular function. Research has indicated that upon activation, P2X7R can further stimulate the NLRP3/Caspase‐1 signaling pathway, resulting in the release of numerous inflammatory factors that may exacerbate the progression of AMD. Furthermore, this activation can lead to an increase in VEGF levels [47]. A study has demonstrated that P2X7R is instrumental in facilitating paracrine communication between retinal cells and microglia, which are likely the first cells to detect ATP expression induced by retinal injury. This process involves the communication between different retinal cell types and ultimately contributes to the progression of AMD [48]. In a mouse model of light‐induced retinal degeneration, it has been shown that P2X7R is overexpressed in damaged retinal tissue, and downregulating P2X7R protein levels can prevent the loss of retinal photoreceptors and cell apoptosis [22].

Interestingly, studies have demonstrated that, compared to wild‐type mice, P2X7 KO mice exhibit diminished phagocytic function in monocytes and macrophages, as well as phenotypic characteristics reminiscent of the early stages of AMD. These characteristics include thickening of Bruch's membrane, loss of retinal pigment epithelial cells, and subretinal inflammation [49]. Subsequent research has revealed a novel biological function of the P2X7R: the regulation of membrane fluidity in leukocytes. This function operates independently of its roles in mediating phagocytosis and pore formation, presenting a promising therapeutic target for late‐stage AMD [50, 51]. This study may elucidate why P2X7 knockout mice exhibit phenotypes that resemble the early stages of AMD. P2X7R plays a complex role in the pathology of AMD by modulating both leukocyte membrane fluidity and phagocytic function. While targeting P2X7R represents a promising therapeutic strategy, several challenges must be addressed, including mechanical complexity, drug selectivity, and clinical translation. Future research should focus on developing specific inhibitors and validating their efficacy in disease models that closely mimic human conditions.

The influx of Ca^2+^ following the opening of P2X7R channels represents a significant consequence. This Ca^2+^ influx may activate apoptotic pathways in neural cells, involving members of the caspase family in P2X7R‐mediated neural cell apoptosis [52]. Carver et al. demonstrated that P2X7R receptor knockout (KO) mice exhibit a reduction in AMD‐like defects and a decrease in the accumulation of microparticles (MPs) due to oxidative stress in Sod1 KO mice [53]. Furthermore, studies have indicated that amyloid‐β peptide (Aβ) induces caspase‐independent apoptosis by activating the P2X7R receptor in human retinal Müller cells. The administration of P2X7R receptor antagonists has been shown to mitigate the toxicity of Aβ to the retina, thereby delaying the progression of AMD [54, 55]. Recent research has highlighted the role of C3 in retinal degenerative diseases. Low levels of complement activation aid microglial phagocytosis and help maintain retinal homeostasis, while excessive activation may contribute to disease progression [56]. Future research could focus on these aspects to explore the mechanisms primarily responsible for AMD and to develop strategies to delay its progression.

In the pathological progression of AMD, the interplay and regulation between the P2X7R and various other molecular entities create a complex network that encompasses inflammation, apoptosis of neural cells, the release of growth factors, and additional factors. This intricate network collectively facilitates the advancement and progression of AMD. Through comprehensive investigations into the functions and interactions of P2X7R and its associated molecules, we can achieve a deeper understanding of the pathogenesis and pathology of AMD. Such insights may lead to novel strategies and methodologies for treating related disorders, including the identification of drug targets for P2X7R and the modulation of growth factor and neurotransmitter release.

### 4.3 Retinitis Pigmentosa

Retinitis pigmentosa (RP) is a genetic ocular disorder characterized by the progressive degeneration of photoreceptors and a concomitant narrowing of the visual field. Despite extensive research, the underlying mechanisms of RP remain partially understood [1, 57, 58]. Recent investigations have indicated that ion channels and signaling pathways, particularly the P2X7R, may play a significant role in the pathophysiology of this condition. The P2X7R, an ion channel essential for retinal function, may influence retinal health and is implicated in the onset and progression of retinal pigmentary degeneration [59]. P2X7R is expressed in retinal pigment epithelial cells and is known to regulate inflammatory responses and apoptosis within retinal tissues. Dysregulation of P2X7R expression and function in the context of retinal pigmentary degeneration may exacerbate inflammatory processes and increase apoptotic activity, thereby contributing to the progression of the disease [60]. Recent studies have demonstrated that the P2X7R/CX3CL1/CX3CR1 signaling pathway is involved in a chemically induced RP rat model. Inhibition of this pathway has been shown to mitigate the overactivation of microglia and the subsequent release of inflammatory cytokines, which in turn alleviates photoreceptor degeneration associated with RP [1]. Furthermore, research conducted by Martínez‐Gil et al. has revealed elevated expression of P2X7RR in the rd10 mouse model of RP. These findings suggest that P2X7R‐mediated neuroinflammation plays a critical role in the progression of the disease and is associated with vision loss in patients with RP [23]. The activation of P2X7R can initiate intracellular signaling cascades that modulate various molecules and enzymes involved in inflammation, apoptosis, and oxidative stress. These signaling pathways may disrupt normal retinal cell function, thereby contributing to the development of retinal pigmentary degeneration.

Dysregulation of P2X7R expression and function may result in abnormalities in these processes, leading to increased inflammation and apoptosis in retinal cells. Such dysregulation may contribute to the progression of retinal pigmentary degeneration [61]. The involvement of P2X7R in retinal pigmentary degeneration is mediated through multiple pathways. Inflammation, a critical component of these pathways, is initiated by the activation of P2X7R, which leads to the release of inflammatory mediators that further exacerbate the inflammatory response. Apoptosis, another essential pathway, is also activated by P2X7R, resulting in the death of retinal cells. Collectively, these processes culminate in damage to retinal pigment cells and the subsequent progression of the disease [62].

Silverman et al. discovered that multiple complement components were significantly upregulated in the retinas of individuals with RP and in the rd10 mouse model. Further experiments demonstrated that the C3‐CR3 signaling pathway is a key regulator of the interaction between microglia and photoreceptors [63]. Additionally, studies have shown that oxidative stress plays a significant role in RP. The activation of P2X7R leads to the production of a substantial amount of ROS [64, 65] and also triggers microglial activation. In the early stages of RP, microglial activation facilitates the clearance of cellular debris in the retina, thereby contributing to retinal homeostasis. However, in the later stages of the disease, sustained inflammation and ROS production can accelerate disease progression. This indicates that P2X7R‐induced microglial activation may have a dual role in RP [66]. Consequently, the complexity of the various adaptive and detrimental roles of microglia in retinal diseases may necessitate interventions aimed at achieving precise modulation rather than broad and complete inhibition, to attain optimal therapeutic outcomes.

Furthermore, genetic mutations and other hereditary factors may contribute to the pathogenesis of retinal pigmentary degeneration. Mutations in the P2X7R gene, along with other associated genes, can disrupt the normal functioning of P2X7R, resulting in its dysregulation and potentially exacerbating the disease process. To further elucidate the role of P2X7R in retinal pigmentary degeneration, additional research is necessary to investigate the molecular mechanisms that underlie inflammation, apoptosis, and other relevant pathways.

## 5 Genetic Variations in P2X7R

The genetic variations of P2X7R can significantly influence disease risk and susceptibility. Gain‐of‐function variants may lead to the overactivation of P2X7R, triggering excessive inflammatory responses or cell death, thereby increasing susceptibility to neurodegenerative and autoimmune diseases. It has been reported that the minor allele variants rs1718119 and rs2230912, when acting in concert, enhance receptor function, contributing to a pro‐inflammatory state associated with inflammatory diseases such as rheumatoid arthritis, Sjögren's syndrome, and systemic juvenile idiopathic arthritis [67]. Conversely, loss‐of‐function variants may diminish P2X7R activity, resulting in reduced inflammatory responses or cell death, which may confer protection against certain diseases. An Australian case‐control cohort study found that a rare loss‐of‐function, low‐frequency allele of the P2X7 receptor single‐nucleotide polymorphism (SNP) rs28360457 is significantly associated with a protective effect against multiple sclerosis [68]. However, this variant may also disrupt the normal functioning of the immune system. Certain rare haplotypes, such as the combination of P2RX7 Gly150Arg and P2RX4 Tyr315Cys, can significantly alter P2X7R function, impairing the phagocytic ability of macrophages or microglia and thereby increasing susceptibility to diseases such as AMD [69].

Genetic variants of the P2X7R may serve as biomarkers for specific diseases, facilitating early diagnosis and risk assessment. The development of agonists or antagonists targeting P2X7R could offer novel strategies for treating related conditions. Although research on retinal diseases associated with mutations in the P2X7R gene is limited, this area may become a key focus for future studies. By integrating genomics, transcriptomics, and proteomics, we can conduct a comprehensive analysis of the functional impact of P2X7R gene variants and their roles in disease. Furthermore, personalized prevention and treatment strategies could be developed based on an individual's P2X7R genotype. Additionally, exploring the common and specific roles of P2X7R gene variants across different diseases may yield valuable insights.

## 6 Potential Therapies Targeting P2X7R for Retinal Diseases

P2X7R is implicated in the pathogenesis and progression of various diseases, including retinal disorders. Recent research has focused on its potential therapeutic role in managing retinal diseases (Table 1). Kong et al. demonstrated that 3TC, a nucleoside reverse transcriptase inhibitor, can concurrently decrease P2X7R levels in STZ‐induced mice, thereby mitigating pyroptosis, apoptosis, and high‐glucose‐induced retinal damage. Additionally, it was shown to alleviate damage to retinal endothelial cells induced by high glucose and lipopolysaccharides (LPS) In Vitro [19]. Yang et al. reported that H3 relaxin inhibits apoptosis in HRMECs and the release of inflammatory mediators triggered by AGE. This effect is mediated through the inhibition of the P2X7R/NLRP3 signaling pathway [33]. Furthermore, dihydrotanshinone has been identified as a novel inhibitor of P2X7R, with studies evaluating its protective effects against high glucose (HG) and BzATP‐induced damage in an In Vitro BRB model comprising retinal pericytes, astrocytes, and endothelial cells. Treatment with dihydrotanshinone was found to preserve BRB integrity from HG/BzATP‐induced damage and to reduce ROS production [39]. Additionally, a study indicated that the inhibitor JNJ47965567 effectively mitigates the detrimental effects of high glucose on human pericytes [70]. The findings indicate that P2X7 receptor inhibitors, specifically A740003 and AZ10606120, provide protective effects against hyperglycemia‐induced damage in HREC by preventing lactate dehydrogenase (LDH) release and safeguarding the BRB. This intervention significantly diminishes hyperglycemia‐induced expression and release of IL‐1β [71]. Research conducted by Clapp et al. demonstrated that the blockade of P2X7R in STZ‐induced diabetic rats effectively reversed the increased retinal vascular permeability, VEGF accumulation, and expression of IL‐6, without affecting blood glucose levels. The authors proposed that P2X7 receptor blockade may represent a viable therapeutic strategy for addressing early microvascular alterations associated with DR [28].

**Table 1:** List of the potential agents of P2X7R in retinal diseases.

Disease	Agent	Target	Pathway	Effect	Animal/cell model	Reference	Limitation
Diabetic retinopathy	3TC (transcriptase inhibitor lamivudine)	P2X7R	↓P2X7R, NLRP3	Protective	C57BL/6J mice/mouse retinal endothelial cells	[19]	No elucidate P2X7 from an ion channel to a pore
	3TC	P2X7R	↓P2X7R	Protective	C57BL/6J mice diabetes	[72]	NO experiment in cell
	H3 relaxin	AGE	↓P2X7R, NLRP3	Protective	Human retinal microvascular endothelial cells/Rat	[33]	Mechanism not discussed in depth
	Dihydrotanshinone/JNJ47965567	P2X7R	↓ROS/P2X7R/ZO‐1	Protective	Human retinal endothelial cells, pericytes, and astrocytes	[39]	NO experiment In Vivo
	AZ10606120/A740063	P2X7R	↓P2X7R, VEGF	Protective	Wistar rats	[28]	Mechanism not discussed in depth
	JNJ47965567	P2X7R	↓P2X7R/IL‐1β	Protective	Human retinal pericytes	[70]	Experimental methods lack diversity
	A438079	P2X7R	↓CD40, P2X7R	Protective	Human RECs and Muller	[31]	NO experiment In Vivo
	AZ11645373/A438079	P2X7R	↓P2X7R, P2X4R	Protective	HUVECs	[36]	NO experiment In Vivo
Age‐related macular degeneration	Berberine	P2X7R	↓P2X7R, TNF‐α,	Protective	P2X7R KO mice/C57BL/6J mice	[22]	No experiment in cell
	3TC	P2X7R	IL‐1β/	Ineffective	HIV infected patients	[3]	Uncertainty in intraocular drug concentration
	A438079/AZ10606120	P2X7R	↓P2X7R, Membrane fluidity	Deleterious	patients with AMD/P2X7 KO mice	[50]	Not clear molecular pathways
	/	/	P2X7R	Deleterious	P2X7‐null mice	[49]	No experiment in cell
	A740003	P2X7R	↓P2X7R, IL‐1β, VEGF	Protective	ARPE‐19/C57BL/6 mice	[47]	Not clear direct interaction of P2X7R and NLRP3
	A740003	P2X7R	↓P2X7R, NF‐KB, NLRP3	Protective	P2X7R KO mice /Human and mouse RPE	[46]	Experimental methods lack diversity
Age‐related macular degeneration	A740003	P2X7R	↓ROS	Protective	Human ARPE‐19	[73]	NO experiment In Vivo
	Saffron	P2X7R	↓P2X7R, NF‐KB, NLRP3		Mouse retinal photoreceptor‐derived 661W cell	[6]	NO experiment In Vivo
Retinitis pigmentosa	Bujing Yishi tablets	P2X7R	↓P2X7R, CX3CL1, CX3CR1	Protective	MNU‐induced RP rats,	[1]	Complement molecules should be explore
	pyridoxal‐phosphate‐6‐azophenyl2′,4′‐disulfonic acid (PPADS)	P2X7R	↓P2X7R	Protective	Rats and rd1 mice	[62]	Mechanism not discussed in depth
	Brilliant blue G	P2X7R	P2X7R, caspase‐8	Protective	P2X7R KO mic and mouse primary retinal cell e	[60]	Only part of the mechanisms

Berberine (BBR) has been shown to reduce the overexpression of P2X7 receptors in retinal ganglion cells and Müller cells in a mouse model of AMD. BBR also mitigated retinal light damage by decreasing histological injury, cell death, and inflammatory responses [22]. Additionally, Bujing Yishi tablets (BJYS), a traditional Chinese herbal remedy, have been reported to slow the progression of RP by inhibiting the P2X7R/CX3CL1/CX3CR1 signaling pathway [1]. A recent retrospective clinical study found no statistically significant differences in the prevalence of early‐to‐intermediate AMD, geographic atrophy, or exudative AMD between HIV patients who utilized P2X7 receptor inhibitors and those who did not [3]. Consistent with previous animal studies, the result showed no statistically significant differences, a possible reason for the dosage may be that it is inadequate to reach an effective P2X7R inhibitory concentration in the retina. Furthermore, HIV patients often display elevated levels of inflammation and immune system abnormalities, which may independently affect the progression of ARMD, irrespective of the role of P2X7R.

## 7 Challenges in the Development of P2X7R Inhibitors

P2X7R inhibitors have demonstrated promising potential in various animal disease models, particularly in the contexts of neuroinflammation and neurodegenerative diseases; however, their clinical application faces significant challenges. Notable differences exist between animal models and humans, complicating the translation of preclinical findings to clinical settings. Many P2X7R inhibitors also exhibit off‐target effects by inhibiting other P2X receptor subtypes, which may lead to unintended consequences. Additionally, some P2X7R inhibitors suffer from low stability and bioavailability In Vivo, limiting their clinical utility. Given that P2X7R plays a role in normal physiological processes, long‐term inhibition may result in unforeseen side effects. Furthermore, the mechanisms of P2X7R action may vary across different diseases, indicating that a single inhibitor may not be universally applicable to all indications.

To address these challenges, future efforts should concentrate on developing highly selective inhibitors through structural optimization and drug design. Combining P2X7R inhibitors with other therapeutic approaches, such as anti‐VEGF agents or antioxidants, may enhance treatment efficacy. Furthermore, screening patient populations based on P2X7R activity or genetic profiles could facilitate personalized treatment strategies. Despite these promising avenues, significant obstacles remain, and the journey toward the clinical application of P2X7R inhibitors is still lengthy and intricate.

## 8 Conclusion

**Figure 1:**
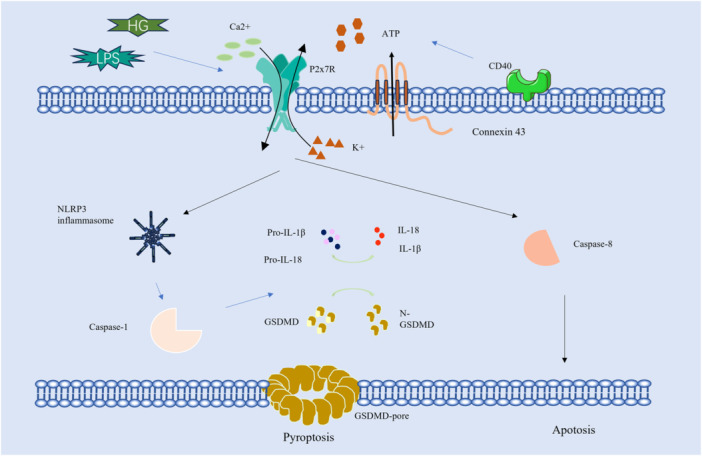
Molecular signaling pathway of P2X7R in retina.

In summary, the investigation of P2X7R in relation to retinal diseases holds significant scientific and clinical importance. P2X7R is a vital component of the P2X receptor family, playing a key role in various physiological processes, particularly in immune regulation and cellular signaling within the retina (Figure 1). A comprehensive understanding of the diverse functions of P2X7R in both health and disease contributes to our knowledge of its potential as a therapeutic target. Future research initiatives centered on P2X7R may provide valuable insights into novel treatment strategies for a variety of conditions, including inflammatory diseases and retinal disorders.

## Author Contributions

**Chunli Li:** writing – original draft, and funding acquisition. **Binsheng Wang:** conceptualization, writing – review and editing, supervision.

## Ethics Statement

The authors have nothing to report.

## Consent

The authors have nothing to report.

## Conflicts of Interest

The authors declare no conflicts of interest.

## Data Availability

The authors have nothing to report.
